# Febris Nigra—“Black Death”

**Published:** 1866-08

**Authors:** 


### Febris Nigra—"Black Death."

A singular and apparently hitherto unknown disease has recently made its appearance in the city of Dublin, the cases so far proving uniformly and rapidly fatal. The first case occurred on the 19th of March, and up to the close of May four others had been reported. The subjects attacked were young persons, and apparently in the most perfect health, and the duration of the disease varied from eleven to seventy-two hours. The essential characters of the disease may be gathered from the following comparisons drawn by Dr. Lyons, in his clinical lectures, between it and those diseases to which it presents the most marked resemblances.—Technically defined, Dr. Lyons regards the disease as the algid stage of an essential zymotic febrile condition, the nature of which is as yet undetermined.

*Comparison with Yellow Fever.*—In the depression of the circulation and deep livid discoloration present in both diseases will be at once seen a resemblance sufficiently remarkable between the algid form of yellow fever and the Irish "black death." In the clearness of the intellect and the undisturbed state of the faculties will be found another point of resemblance. On the other hand, in the absence of yellow coloration before or after death in the conjunctivæ or other parts in the cases of "black death," and in the presence of hemorrhages from various parts of the mucous surfaces in yellow fever, as well as the striking concomitant of black vomit, must be noticed very essential points of difference.

*Comparison with Cholera.*—In contrasting the phenomena of the malady in question with certain of those presented in cholera, Dr. Lyons is of the opinion that even still less pathological affinity can be traced than between yellow fever and black death, or febris nigra, which may form no inappropriate designation of the disease. In the *cholera sicca*, to which alone, in his opinion, the Irish black death can be compared, there is, it is true, an absence of vomiting and purging, and so far a similarity; but the dry cholera is attended with muscular cramps and abdominal pains, and the discoloration of the surface is essentially that of minute venous congestion and not that of cutaneous transudation of blood, and after death muscular rigidity is extreme. In cholera, likewise, the voice is often reduced to a whisper, the eye sunk, the nose pinched, the hands and fingers shrunken, while, with the absence of radial pulse, some of the most remarkable of the cases of "black death" exhibited not only full possession of the faculties, but perfect voice and distict articulation.

*Comparison with Typhus.*—On reviewing carefully all the phenomena presented by the cases of black death hitherto observed, Dr. Lyons fails to find any support for the opinion which would regard them as any form of typhus fever. The insidiousness of the invasion with the early depression of the circulating system might be considered to establish a faint resemblance; but in the perfect possession of the faculties and distinctness of speech retained to within a short time of the fatal issue by the patients in some of the most marked cases of the black death, will be found characters which point to an essential difference from the typhus state, in which stupor is a leading and necessary feature. Hence Dr. Lyons dismisses the idea that the malady in question is to be ranked under any form of typhus, or that it has any true pathological affinity to any phase or stage of that morbid state.

Dr. Lyons is disposed to look at the meteorological condition of the atmosphere for an explanation of the occurrence of these remarkable cases, but no satisfactory conclusion is yet arrived at concerning them. The fatal issue was rapid to a degree not often equaled in the most severe epidemics on record, and he is disposed to conclude that the occurrence of these cases of such unusual character and uniform fatality points to the possible visitation of some epidemic of appalling severity and no ordinary character.—*N. Y. Medical Journal.*

